# Dysautonomia and Postural Orthostatic Tachycardia Syndrome (POTS) in the ENT Clinic: Differentiating Orthostatic Dizziness From Vestibular Migraine and Persistent Postural-Perceptual Dizziness (PPPD)

**DOI:** 10.7759/cureus.108903

**Published:** 2026-05-15

**Authors:** Philip Zitser, Ilana Kolomiyets, Maheen Imran, Aashi Kulkarni, Jenika Patel, Nina St. Martin, Ahmad Ahmadi

**Affiliations:** 1 Medicine, New York Institute of Technology College of Osteopathic Medicine, Old Westbury, USA; 2 Medicine, Nova Southeastern University Dr. Kiran C. Patel College of Osteopathic Medicine, Fort Lauderdale, USA; 3 Anatomy/Neuroanatomy/Dentistry, Nova Southeastern University Dr. Kiran C. Patel College of Osteopathic Medicine, Fort Lauderdale, USA

**Keywords:** benign paroxysmal positional vertigo (bppv), dysautonomia, orthostatic dizziness, persistent postural-perceptual dizziness (pppd), postural orthostatic tachycardia syndrome (pots), vestibular migraine

## Abstract

One of the most common complaints in ENT clinics is dizziness. Although most cases are caused by vestibular neuritis and benign paroxysmal positional vertigo (BPPV), a significant number of patients experience non-vestibular dizziness. One non-vestibular cause is postural orthostatic tachycardia syndrome (POTS), which presents with symptoms similar to vestibular migraine (VM) and persistent postural perceptual dizziness (PPPD). Dizziness from POTS is related to dysautonomia rather than the vestibular system. In otolaryngology, understanding the autonomic nature of non-vestibular dizziness is important for accurate diagnosis. This study aims to review the clinical characteristics and pathophysiology of POTS, vestibular migraine, and PPPD; examine historical, physical, and diagnostic findings that differentiate autonomic and vestibular causes of dizziness; and develop a more specific model for evaluating chronic dizziness in the ENT setting. Literature on dizziness, vestibular and autonomic pathophysiology, and the clinical presentation of POTS, VM, and PPPD is reviewed in this paper. The timing of symptoms, their triggers, and objective assessment are key differentiating factors for these disorders. Various diagnostic tools, including orthostatic vital signs, oculomotor assessment, gait and balance evaluation, vestibular function assessment, tilt table testing, and neuroimaging, are also reviewed. Clear distinctions among POTS, VM, and PPPD are made.

The diagnostic criteria for POTS are orthostatic tachycardia and posture-dependent symptoms that improve when the patient is in a recumbent position, effectively ruling out vestibular involvement in these patients. Vestibular migraine presents with vertigo accompanied by migraine features such as photophobia, phonophobia, or headache, typically triggered by sensory or environmental factors rather than changes in posture. PPPD manifests as chronic non-spinning dizziness and imbalance lasting at least three months, exacerbated by motion, upright posture, and complex visual environments. Orthostatic vital signs and autonomic assessment should be included in the evaluation of patients presenting with dizziness to differentiate between these disorders. Both vestibular and autonomic dysfunction can lead to chronic dizziness. By incorporating autonomic assessments into vestibular evaluations, ENT physicians can apply a more precise diagnostic model for their patients. Identifying dysautonomia-related dizziness, including POTS, helps reduce the misdiagnosis of vestibular disorders. This approach enables physicians to provide more effective interventions for patients with complex dizziness.

## Introduction and background

Although a common vestibular complaint, dizziness is among the most frequent presenting symptoms in otolaryngology, affecting 15-20% of adults annually in general medical settings [1]. Dizziness is a broad term that can encompass various neurological sensations, including vertigo (a spinning sensation), lightheadedness, imbalance, or a sense of disorientation. This statistic not only quantifies its prevalence but also underscores the diagnostic responsibility of physicians. The high frequency of patients presenting with dizziness increases the risk of a “rule-of-thumb” diagnosis, where common vestibular etiologies are presumed before alternative causes are fully evaluated. Although most cases are due to peripheral vestibular disorders such as benign paroxysmal positional vertigo (BPPV) and vestibular neuritis, a significant number of patients present with non-vestibular dizziness [2]. Fife challenges the assumption that dizziness always correlates with vestibular pathology, emphasizing that symptom location does not necessarily indicate its origin [2].

Non-vestibular dizziness is often misdiagnosed, especially when symptoms are chronic, non-specific, and respond poorly to standard vestibular treatments. Patients with persistent postural-perceptual dizziness (PPPD) experience non-spinning dizziness that is exacerbated by upright posture, active or passive movement, and complex visual stimuli [3]. Staab et al. characterize PPPD as a disorder of dysfunctional sensory integration - involving visual, spatial, and proprioceptive systems - rather than structural vestibular damage, shifting the focus to central processing mechanisms. Vestibular migraine (VM), one of the most common causes of episodic vertigo, induces visually- or head motion-triggered dizziness, or persistent dizziness, making it difficult to distinguish from motion sickness and PPPD [4]. Lempert et al.'s research further shows that underlying pathology can amplify sensory responsiveness and create symptom overlap that does not align with traditional diagnostic criteria. Clinical overlap between autonomic disorders, such as PPPD and VM, highlights the diagnostic ambiguity in ENT practice. Rather than representing entirely distinct pathologies, these conditions may exist on a spectrum of sensory and autonomic dysregulation.

Dysautonomia syndromes, such as postural orthostatic tachycardia syndrome (POTS), are gaining recognition as common causes of orthostatic intolerance and chronic dizziness. Dysautonomia refers to a dysfunction of the autonomic nervous system, which regulates involuntary processes such as heart rate and blood pressure. This recognition frames vestibular symptoms as potentially secondary to cardiovascular autonomic instability. POTS criteria include an increased heart rate (≥30 bpm) within 10 minutes of standing without orthostatic hypotension (a blood pressure drop >20/10 mmHg). In addition to tachycardia, POTS patients present with palpitations, lightheadedness, presyncope, mental clouding, and fatigue [5]. Rather than considering cognitive and fatigue-related symptoms as secondary manifestations of anxiety or functional disorders, Raj’s clinical description suggests viewing them as central diagnostic features [5]. Dizziness, imbalance, and brain fog are often misdiagnosed as symptoms of vestibular conditions. Without an orthostatic vital assessment, the underlying cause of these symptoms remains unidentified, leading to inaccurate diagnoses.

Without examining autonomic symptoms that cause dizziness, appropriate treatment may be delayed, resulting in prolonged patient illness. This not only increases symptom burden but also contributes to patient frustration and excessive healthcare utilization. The objective of this review is to provide ENT clinicians with a differential diagnostic framework capable of distinguishing orthostatic dizziness related to dysautonomia from VM and PPPD. By integrating autonomic evaluation into routine dizziness assessment, clinicians can move away from symptom-based categorization and adopt a more physiologically precise diagnostic approach.

## Review

Definitions and phenomenology

Dizziness Subtypes

Vertigo: Vertigo is the illusion of environmental motion, usually described as “spinning” or “whirling”. It is usually a neurologic or inner-ear problem, causing patients to feel imbalanced, nauseous, and can illicit vomiting. The sense of motion is usually rotatory, but it may be more linear, comparable to being on a boat at sea. Vertigo causes disorientation in space and a sense of illusory motion, reflecting dysfunction in the vestibular system [6].

Disequilibrium: Disequilibrium is a disturbance in balance or coordination that impairs ambulation, typically due to inner ear issues, aging, or neurologic factors. Patients commonly describe a problem with their legs and feeling dizzy in the head. Patients with disequilibrium all have the perception that their ambulation is the root cause of the problem; thus, an observation of the patients’ gait alongside a neurologic examination is essential in evaluating disequilibrium [6].

Lightheadedness: Lightheadedness is often difficult for patients to describe apart from feeling dizzy. It is caused by a temporary reduction in blood or oxygen to the brain from dehydration, sudden position changes, stress, or low blood sugar. The sensation is not vertiginous or presyncopal, and is a sensation in the head rather than ambulatory. Some patients with lightheadedness describe feeling like they’re floating or that their head is not attached to their body, and symptoms improve with lying down [6].

Presyncope/orthostatic dizziness: Presyncope/orthostatic dizziness is a sensation of impending loss of consciousness for a few seconds to a few minutes. It is caused by a temporary reduction in blood flow to the brain, usually due to dehydration or standing up too fast. Patients describe feeling as if they’re about to pass out, but most do not. Lightheadedness, dizziness, nausea, vomiting, and blurred vision are symptoms that patients commonly present with [6].

Review of key syndromes

Postural Orthostatic Tachycardia Syndrome (POTS)

POTS is a dysautonomia that presents with an increased heart rate of at least 30 beats per minute within 10 minutes of standing or tilting the head up, without orthostatic hypotension [7]. Patients with POTS experience orthostatic intolerance symptoms that include lightheadedness, presyncope, palpitations, fatigue, cognitive impairment, and exercise intolerance. Symptoms depend on posture and improve when lying down. The cause of POTS is complex but includes partial autonomic neuropathy, hypovolemia, hyperadrenergic states (elevated plasma norepinephrine levels), and impaired venous return [5]. Because patients usually present with dizziness and imbalance without true vertigo and have normal vestibular testing, POTS is often misdiagnosed as a VM or a chronic functional vestibular disorder.

Vestibular Migraine (VM)

VM is a common cause of episodic vertigo, which is defined by recurrent vestibular symptoms of moderate-severe intensity lasting five minutes to 72 hours in patients with a current or prior history of migraine. At least half of vertigo episodes must have at least one feature of headache with migraine characteristics, photophobia, phonophobia, or aura [4]. VM affects approximately 1% of the general population, with 10% of patients with migraine suffering from VM [8]. Unlike orthostatic dizziness in POTS, VM episodes are not strictly posture-dependent and are often triggered by typical migraine inducers such as stress, hormone fluctuations, or sleep disruption.

Persistent Postural-Perceptual Dizziness (PPPD)

PPPD is a functional vestibular disorder characterized by constant non-spinning dizziness and unsteadiness on over half the days during a 3+ month timeline. Symptoms are exacerbated by upright posture, active or passive motion, and exposure to complex visual stimuli such as busy environments or scrolling screens. PPPD is frequently caused by an acute vestibular event, VM, medical illness, or psychological stressor [3]. Rather than a true rotational vertigo, patients commonly present with a rocking or swaying sensation. Although symptoms worsen with upright posture, PPPD does not involve orthostatic tachycardia, distinguishing it from POTS. Association with migraine and anxiety disorders is common.

Pathophysiology

Baroreflex, Cerebral Autoregulation, and Vestibulo-Autonomic Integration

The body's coordinated interaction between the vestibular system and the autonomic nervous system is fundamental for responding to postural changes. This coordination explains why patients experience dizziness and presyncope. When a person stands up, gravity immediately affects the blood returning to the heart. This can briefly reduce cerebral perfusion pressure, increasing the risk of presyncope. The baroreceptor reflex, a system that acts as a buffer to these hemodynamic shifts, senses arterial stretch at the carotid sinus and aortic arch. It then rapidly adjusts autonomic signals [9]. Signals from the baroreceptors travel through the glossopharyngeal and vagal nerves to the nucleus tractus solitarius.

These signals adjust heart and blood vessel activity to restore pressure and flow [9]. If blood pressure falls, baroreceptor activity falls, and the vagal effect on the heart decreases. At the same time, sympathetic activity increases, causing a higher heart rate, stronger contractions, and increased blood vessel resistance [9]. In dysautonomia, these compensatory responses may be delayed or inadequate, leading to symptoms often reported as "dizziness" in clinical settings [10]. Understanding how these systems interact helps clinicians distinguish between presyncope and vestibular vertigo during the examination of patients undergoing an ear, nose, and throat evaluation.

Cerebral autoregulation stabilizes cerebral blood flow during brief changes in blood pressure by altering cerebrovascular resistance. This adaptation protects the brain from pressure drops when standing. Notably, blood pressure in the arm may remain normal while brain blood flow decreases, especially in people with low cerebrovascular reserve or weakened reflexes [11]. Research on orthostatic intolerance shows that if brain blood flow drops too much during standing, people can feel lightheaded, experience blurred vision, or have reduced cognitive function, even if blood pressure remains normal [11]. Additionally, problems in autonomic or cerebral regulation in dysautonomia can worsen dizziness and lead to a 'cerebral perfusion phenotype' that mimics vestibular dysfunction [10]. Furthermore, neurovascular coupling adjusts blood flow in response to the brain’s metabolic needs during cognitive effort and sensory processing, not just to pressure changes [11]. Thus, managing blood pressure and balance relies on the heart-brain connection and directly affects symptoms when standing.

Vestibulo-autonomic integration is essential for regulating both balance and blood pressure during movement. This association links the inner ear and autonomic reflexes that affect heart and vascular function during head and body motion. The vestibulosympathetic reflex works alongside the baroreceptor reflex by generating sympathetic adaptations during movement, thus supporting venous return and preserving arterial pressure during postural changes [12]. Pathways from the vestibular nuclei to brainstem centers coordinate balance, ocular, cervical, and cardiovascular reflexes during daily activity [13]. This integrated system explains why dizziness may present as faintness or motion sensitivity, depending on the involved reflex pathways [13].

Recent research suggests that vestibular-autonomic connections are implicated in orthostatic dizziness and the overlap of vestibular and autonomic disorders, which is clinically pertinent for ear, nose, and throat settings where chronic dizziness is evaluated [14]. In practice, this merging clarifies why symptoms may worsen with both upright posture and head movement, even if vestibular assessments are normal [14]. Therefore, balance and hemodynamic control are closely linked via vestibular input during daily activities.

*Postural Orthostatic Tachycardia Syndrome* (*POTS) Mechanisms*

The various mechanisms underlying POTS result in varied symptom triggers and presentations. Frequent overlap of mechanisms in patients adds to its complexity, as such overlap shapes symptom triggers in different contexts. For example, one hypothesized mechanism in POTS is excessive sympathetic activity, which provokes tachycardia and presyncopal symptoms upon standing. In this type of POTS, the body releases excessive catecholamines immediately after standing, causing palpitations, tremors, and "adrenergic surges" that may be misdiagnosed as anxiety or cardiac arrhythmias [10].

For some individuals, this sympathetic response represents the primary problem; however, for many others, it is an autonomic response to low blood volume or inadequate peripheral vasoconstriction. As a result, the heart increases its rate to maintain sufficient blood flow [9]. This distinction is important because a rapid heart rate during standing can have several causes, each needing different treatments [10]. In ear, nose, and throat clinics, individuals may report "dizziness," but this is often presyncope with high autonomic arousal rather than true vertigo. Mechanistically, excessive sympathetic activity results in overactive reflexes during standing, bringing about increased symptoms and avoidance behaviors [9]. Thus, excessive sympathetic activity is only one factor in POTS.

Hypovolemia is common in POTS and plays a central role in developing a rapid heart rate, even with normal blood pressure. When there is less blood in the vessels, the heart fills and pumps out less with each beat. As a result, the body compensates by elevating heart rate when standing to maintain blood flow to the brain and other organs [10]. An important aspect of this compensation is the renin-aldosterone paradox. Hormonal levels that usually maintain blood volume are inappropriately low for the degree of volume loss, showing a defect in renal hormone regulation rather than simple dehydration [15].

This observation supports salt and fluid supplementation in some cases, because some individuals have reduced intravascular volume despite sufficient hydration [16]. Recent research further supports hypovolemia as a primary contributor to symptom severity and rapid heart rates in POTS [17]. In ENT practice, it is important to recognize that hypovolemia can worsen symptoms in the setting of heat, illness, or reduced fluid intake. Although these symptoms may mimic vestibular dysfunction, hypovolemia is often the primary cause. Therefore, identify hypovolemia as a modifiable factor that can worsen POTS symptoms.

In addition to hypovolemia, poor venous return markedly contributes to POTS by causing excessive pooling of blood in the lower extremities and the splanchnic circulation during upright posture. This pooling, due to impaired peripheral vasoconstriction, reduces central blood volume and stroke volume, ultimately triggering compensatory tachycardia. When blood vessels fail to constrict adequately, blood accumulates in the legs and abdominal cavity, reducing cardiac output and leading to a rapid heart rate [10].

Although arm blood pressure may remain normal, pooling can reduce central blood volume and decrease cerebral blood flow during prolonged standing. Consequently, treatments like compression stockings and physical maneuvers that improve venous return can relieve symptoms and raise standing tolerance [5]. Poor venous return frequently coincides with hypovolemia and excessive sympathetic activity, forming a vicious cycle in which pooling triggers a rapid heart rate and amplifies awareness of symptoms and fatigue [9]. In clinical practice, poor venous return explains why dizziness worsens with prolonged standing, rather than dizziness related mainly to head movement. Thus, poor venous return is an important factor in the development of POTS.

Beyond the circulatory factors already described, POTS can also present with symptoms related to neurovascular coupling abnormalities, as well as overall cerebral perfusion dysregulation, especially when POTS patients report "brain fog," blurred vision, or cognitive exhaustion. Neurovascular coupling describes the relationship between neuronal metabolic demand and cerebral blood flow. Disruptions in this relationship can lead to patients perceiving orthostatic stress as being much worse. Blood pressure reviews of cerebral blood flow in orthostatic intolerance indicate that cerebral blood flow or flow velocity can decrease excessively when patients with POTS stand upright, while their blood pressure remains relatively stable [11].

This evidence supports a model of cerebral perfusion regulation of dizziness and cognitive symptoms in response to orthostatic stress, rather than a solely vestibular mechanism. This also provides a physiological basis for how symptoms can worsen in settings that require greater cerebrovascular control, e.g., in visually complex or cognitively taxing environments, or while standing [11]. From an ENT perspective, this mechanism offers a rationale for why some patients appear "vestibular" in relation to visually mediated worsening but have a primary orthostatic physiology that requires evaluation [14]. Therefore, neurovascular coupling abnormalities should be described as a cerebral-perfusion pathway that produces multisystem symptom profiles in POTS.

Vestibular Migraine Mechanisms

VM is commonly understood to be migraine pathophysiology demonstrated through vestibular symptom networks, with trigeminovascular activation as an essential mechanism during attacks. The activation of trigeminal afferents stimulates brainstem pathways that release vasoactive neuropeptides, including calcitonin gene-related peptide (CGRP), which can modulate neural excitability and sensory processing in areas that communicate with the vestibular nuclei [18-19]. This mechanism yields a rationale for why vertigo or dizziness caused by head motion may occur in conjunction with or independent of headache, and why photophobia, phonophobia, and nausea often accompany vestibular symptoms [4].

As narrative syntheses increasingly illustrate, VM is a disorder of network-level dysregulation involving trigeminal, vestibular, and autonomic brainstem circuitry rather than a peripheral lesion in the inner ear [20]. The literature on treatment response confirms the notion of shared migraine circuitry, as several systematic reviews have reported that CGRP-targeted therapies benefit a subset of patients with VM [21]. Therefore, from an ENT perspective, this model confirms the notion that episodic vertigo with migraine characteristics is centrally mediated rather than structurally labyrinthine. Trigeminovascular activation delivers a coherent mechanistic rationale for the vestibular symptoms in VM.

VM can also be defined as a disruption in sensory integration, in which the relative contributions of vestibular, visual, and somatosensory inputs are variable and do not remain constant, resulting in instability in motion tolerance and visually induced dizziness. Neurophysiological and imaging studies show abnormal processing in multisensory hubs, specifically thalamic circuits, during vestibular stimulation, indicative of aberrant gating of vestibular signals and their integration with nociceptive pathways [22]. Connectivity research also implicates temporo-parietal regions involved in vestibular perception and multisensory integration, and supports a network-based mechanism of vulnerability to dizziness in visually complex or motion-rich environments [23].

Synthesis of the data indicates that migraine-related fluctuations in brainstem-thalamocortical excitability distort the estimation of spatial orientation and sensory reliability, leading to vertigo or "rocking" sensation independent of peripheral vestibular input [20]. This view is supported by the common clinical report of symptom provocation in supermarkets, scrolling screens, or patterned floors, which represent environments that require loading of visual-vestibular integration rather than classical positional vestibular pathways [4]. Documenting environment-driven triggers and multisensory hypersensitivity can therefore help distinguish VM from orthostatic dizziness, which correlates more closely with upright duration. Sensory integration disruption should therefore be presented as a clinically relevant pathway in VM.

VM has been increasingly linked to central sensitization, a condition of increased responsiveness of central pain and sensory networks that can broaden triggers and prolong symptoms between attacks. Recurrent trigeminovascular activation may sensitize second- and third-order neurons within brainstem pathways, increasing sensory gain and causing exaggerated responses to otherwise tolerable stimuli [18]. Research has identified higher levels of central sensitization and interictal sensory hyperresponsiveness in VM populations compared to controls, and supports a fairly extensive sensory amplification phenotype as opposed to isolated vestibular dysfunction [24].

The thalamus is consistently identified as a relay for nociceptive and multisensory signals in migraine, and thalamic dysfunction is thought to contribute to photophobia, allodynia, and generalized sensory intolerance [25]. Clinically, central sensitization connects to overlapping syndromes, as patients with frequent VM episodes may develop chronic dizziness patterns and engage in behaviors similar to those seen in functional dizziness presentations [26]. This system illustrates that the broadening of the trigger and chronicity can result from experience-dependent changes in central sensory processing, rather than being solely a descriptive or psychological label. Central sensitization should therefore be represented as a mechanism that expands symptom triggers and maintains disability in VM.

Persistent Postural-Perceptual Dizziness (PPPD) Mechanisms

PPPD is a functionally based neuro-otologic syndrome that results from a precipitating vestibular, medical, or psychological event leading to maladaptive balance strategies that persist. Patients commonly exhibit a highly vigilant postural style defined by stiffness, cautious movements, and excessive attention to balance. However, this attentive approach to posture paradoxically worsens subjective unsteadiness when patients are standing or walking [3]. Diagnostic criteria for PPPD require that symptoms are exacerbated by upright posture and by active or passive motion; however, in PPPD, maintaining posture is a symptom trigger rather than a stabilizing approach [3].

Mechanism models assert that threat appraisal circuitry interacts with sensorimotor control systems to reinforce a defensive posture, even after the initiating trigger is resolved [27]. Review articles that employ neuroimaging-based approaches support this integrated model by describing aberrant connectivity within emotional, visuo-vestibular, and sensorimotor networks that sustain maladaptive motor responses to typical balance demands [28]. Clinically, this mechanism accounts for why patients will often appear relatively stable on short-term bedside assessments, but exhibit debilitating symptoms that escalate with prolonged periods of upright posture. Maladaptive postural control should therefore be represented as a fundamental mechanism toward maintaining symptoms in PPPD.

PPPD is also very strongly correlated with visual dependence, in which visual information is overly relied upon for spatial orientation and balance. This feature of PPPD is illustrated by the reproducibility of worsening of symptoms within environments that contain moving or complex visual information. This illustrates that visually induced destabilization is meaningful at the mechanistic level [3]. Mechanistic reviews indicate that visual dependence represents both a risk factor for developing PPPD and a maintenance factor, as patients who rely heavily on visual motion cues experience dizziness in supermarkets, on scrolling screens, on patterned floors, and in other visually complicated environments [29].

A unified explanation is that visual motion is interpreted as unpredictable or threatening, thereby stimulating hypervigilant scanning and overcorrecting, which further destabilizes the perception of self-motion [27]. Clinical updates report that PPPD is functionally rather than structurally based, and that visually provoked worsening is more indicative of altered multisensory processing than of continued labyrinthine injury [30]. Studies comparing PPPD and episodic disorders, such as VM, illustrate that the context and pattern of visually-induced dizziness can assist in differentiating PPPD from episodic disorders [31]. Therefore, visual dependence should be represented as a fundamental driver of environment-linked symptom escalation in PPPD for clinicians to view. 

PPPD can also be conceptualized as disrupted sensory reweighting, in which the brain fails to adaptively recalculate the weightings of vestibular, visual, and somatosensory input following a triggering dizziness event. Under normal circumstances, sensory weightings change based on context - down-weighting unreliable cues and up-weighting reliable cues - however, in PPPD, the self-regulating process is said to become "stuck", and continues to render the patient vulnerable to upright posture, motion, and visual complexity [3]. Reviews indicate that this disorder unites previously distinct constructs (visual vertigo and chronic subjective dizziness) into a single multisensory recalibration problem centered on persistent maladaptation in central vestibular processing [32].

Neurobiological reviews also support this central vestibular processing hypothesis by illustrating that altered interactions among emotional, visuo-vestibular, and sensorimotor networks would cause the system to depend more strongly on certain cues (typically visual) and underuse vestibular or proprioceptive input, and continue to produce symptoms despite normal peripheral testing [28]. This system also illustrates why triggers can generalize over time: a fixed weighting strategy expands the range of environments and motions that trigger symptoms [27]. Clinically, impaired sensory reweighting connects the patient's daily-life symptom-exacerbation patterns to a coherent neurobehavioral mechanism, rather than merely an obscure complaint. Therefore, impaired sensory reweighting should be represented as a fundamental mechanism for the maintenance of symptoms in PPPD.

Clinical presentations (ENT-relevant)

Postural Orthostatic Tachycardia Syndrome (POTS) and Dysautonomia

POTS typically presents with an array of symptoms, including those pertinent to orthostatic intolerance as well as non-orthostatic symptoms. Orthostatic symptoms include dizziness, presyncope, and lightheadedness upon standing up [33]. These symptoms are typically relieved by sitting down or recumbency. Patients may report cardiovascular manifestations such as palpitations, dyspnea, and chest discomfort [34]. These symptoms may be attributed to inferior lead alterations on an electrocardiogram (ECG) while the patient is upright; however, these findings do not correlate to the existence of coronary artery disease within those patients [5].

Non-cardiac symptoms commonly include fatigue, brain fog, and weakness [5]. Exercise intolerance may be present, and activities of daily living can worsen symptoms, ultimately resulting in fatigue [5]. Other environmental triggers, such as extreme heat and dehydration, aggravate symptoms of POTS [5]. Early counseling is recommended to avoid such conditions by proactively hydrating and increasing sodium intake. Overall, POTS symptoms reflect autonomic dysregulation rather than a primary vestibular etiology. The cardiovascular component of POTS is clinically important and relevant when it comes to distinguishing between VM and PPPD.

Vestibular Migraine

VM symptoms are similar, yet distinguishable from those of POTS and PPPD. Episodic vertigo, including recurrent episodes of internal and/or external vertigo, may be present in VM patients [35]. Internal vertigo presents with an illusory perception of oneself moving, whilst external vertigo is a sensation in which the patient’s surroundings are falsely interpreted as spinning or flowing [35]. Additional vestibular symptoms include visually induced vertigo, unsteadiness, or head motion intolerance. Duration of symptoms varies, with a patient's report ranging from a few minutes to up to 72 hours [36]. The frequency of an attack is variable by patient as well, with attacks on a daily basis or only once or twice a month [36]. Oftentimes, these attacks are sporadic and spontaneous, but environmental triggers such as sleep, stress, head position, or visual stimuli may induce episodes [37]. Unlike POTS, episodic vertigo is not posture-induced, and unlike PPPD, these vestibular episodes are not constant.

Migraine headache is another important symptom of VM. A detailed headache history is an essential component in the physical exam done upon presentation, as it is part of the diagnostic criteria for VM. It is important to note that VM episodes do not always present with a headache, making diagnosis difficult. A study found that of the patients presenting with VM, 50.8% had a family history of migraine and that episodes were accompanied by headache in 49.6% of patients [38]. As per the International Classification of Headache Disorders (ICHD-3), the diagnostic criteria for VM headaches must include two of the following: unilateral location, pulsating quality, moderate or severe intensity, and aggravation by routine physical activity [20]. Additional associated symptoms listed as part of the ICHD-3 diagnostic criteria include the presence of photophobia or phonophobia. Photophobia is an abnormal sensitivity to light [39], and phonophobia is an abnormal sensitivity and fear of sound [40]. With VM, there is a greater distinction to be noted when comparing PPPD and POTS. With the triggers of light, sound, visual motion, and head position, VM is characterized by episodic and recurrent sensory hypersensitivities.

Persistent Postural-Perceptual Dizziness (PPPD)

PPPD presents with a persistent nonvertiginous dizziness, a key feature in helping diagnose PPPD due to its chronic nature. PPPD patients would have persistent non-vertiginous dizziness for long periods of time, established as present on most days for three or more months [3]. This non-vertiginous dizziness has been described by patients as feelings of swaying, rocking, or bouncing [3]. Furthermore, visual stimuli, which can be stationary or moving, may aggravate dizziness in PPPD patients. However, the mechanisms for this are unknown [41]. Exacerbation of symptoms by upright posture, such as standing or walking, is also reported by PPPD patients [32]. Patients may hold on to others or other fixed objects in order to stabilize themselves while walking and/or standing [3]. PPPD lacks the presence of episodic migraine that is particularly associated with the onset of persistent dizziness [26]. Although upright posture may exacerbate dizziness in both PPPD and POTS, there is no orthostatic tachycardia associated with PPPD. This distinction is critical in avoiding misclassification during diagnosis.

Key diagnostic features

History and Symptom Triggers

Dizziness can be challenging to interpret as a clinical presentation. Obtaining a thorough and accurate history remains vital to differentiating between possible causes and managing symptoms. Symptom triggers generally fit into two categories: orthostatic and positional. Orthostatic triggers occur when a person is rising to a standing position from lying down. This should prompt measurement of orthostatic vital signs to assess for potential POTS or orthostatic hypotension. Positional triggers occur when a person changes head or body position, such as rolling over in bed. These may be common in episodic type dizziness and should prompt the completion of positional tests [42]. The timing and frequency of symptoms can also offer a lot of information in terms of guiding a differential diagnosis. Episodic vestibular symptoms are transient, occurring during multiple recurrent events. These events can be spontaneous or triggered, but generally only last between minutes and hours. Chronic vestibular symptoms are persistent, generally lasting from months to years. These symptoms can be stable or progressive in nature [42]. Timing and triggers should remain the focus of the history since the patient’s description of their dizziness can be misleading in forming a diagnosis.

Physical Exam

Orthostatic vital signs: Orthostatic vital signs are commonly used to diagnose dysautonomia. These include measuring heart rate and blood pressure while a patient is supine and standing [43]. When measuring the blood pressure, it is important for the patient to be lying for a minimum of five minutes prior to standing without sitting in between. This is key to increasing test sensitivity. The patient should also remain standing for at least five minutes prior to taking the blood pressure again [44]. Normally, the carotid baroreceptors sense the drop in blood pressure when a person goes from lying to standing, triggering vasoconstriction. However, this feedback loop could be ineffective, which can lead to orthostatic hypotension. This would be indicated by a drop in systolic blood pressure of more than 20 mm Hg or diastolic blood pressure of more than 10 mm Hg [44]. Heart rate is measured after obtaining each blood pressure measurement [43]. This measurement helps to narrow the differential diagnosis.

Gait and balance: The Tinetti Gait and Balance Test is one of the validated tools to help identify dysautonomia among other conditions that may cause falls. The balance assessment is done first, where the patient is asked to rise from a seated position in an armless chair without using their arms. While standing, the patient moves their feet as close together as possible and is asked to make a 360-degree turn before sitting back in the chair. This test is scored using 10 standardized subsets for a total of 16 points. The gait assessment includes having a patient walk 15 feet at a regular pace and have them walk back to the starting point at a quicker pace. This test is scored using seven standardized subsets for a total of 12 points [45]. While these tests are typically used to evaluate falls in older individuals, they can be effective for highlighting underlying pathology in patients presenting with dizziness.

Oculomotor evaluation: Oculomotor evaluation is crucial for evaluating if the presenting dizziness is a sign of an emergency, such as a posterior cerebral circulation stroke or conditions such as VM [42]. A study had 42% of VM patients had abnormal oculomotor evaluation [46]. The OculoMotor assessment tool standardizes the visual target components for an oculomotor evaluation [47]. This aids in differentiating a normal exam from an abnormal one. To measure smooth pursuits, patients follow a slowly moving target from left to right or vice versa. For the evaluation of saccades, patients are asked to perform 10 repetitions of each (vertical and horizontal).

The near point convergence is a measurement of the minimum distance at which an object becomes double for a patient [47]. To test the vestibulo-ocular reflex, a patient is asked to keep gaze steady during various head movements, such as moving the head from right to left. Patients’ eyes are also observed for nystagmus in either direction. One study found that spontaneous vertical nystagmus was 93% specific for VM as opposed to other directions [48]. If there is positive nystagmus, patients can fixate on an object to see if it extinguishes, which can differentiate between peripheral and central vertigo [42].

Objective Testing

Tilt table test: The tilt table test is a diagnostic procedure to assess possible causes of dysautonomia, including POTS. Patients lie strapped on a bed that tilts to a specific angle between 60 and 80 degrees. Blood pressure and heart rate are monitored throughout the approximately 45-minute test. The tilting is meant to stimulate the change from a lying to a standing position, which can trigger fainting or orthostatic hypotension. The test is most valuable if it reproduces the symptoms a patient is already having [49]. Tilt Table Testing is performed in an electrophysiology lab, and patients must qualify to receive this as a referral test. If the patients’ symptoms do not qualify for this test, they may not receive a concrete diagnosis [50]. This suggests the potential for treatment discrepancies within the umbrella of dysautonomia.

Vestibular function tests (Video Head Impulse Test (vHIT): Vestibular function tests help to determine the function of the semicircular canals and otoliths to assess for common vestibular disorders, such as VM. One study found that 73% of patients with VM had at least one abnormal vestibular function test [46]. The video head impulse test stimulates and quantifies the vestibulo-ocular reflex. The test measures eye and head velocity as the head is manually rotated in different directions, measuring each of the semicircular canals’ function independently [51]. Saccades can be noted on refixation of the visual target [51]. One study noted the frequency of the saccade at 9.3% in patients with unilateral vestibular loss [52]. This highlights the importance of saccade observation in the assessment of the video head impulse test. In differentiating between central and peripheral vestibular dysfunction, one study found that the video head impulse test was 9.87% specific, but only 47.89% sensitive [53]. This indicates that this test may not be preferred to perform on its own. Instead, a combination of vestibular function tests may provide more clinical utility.

Vestibular function tests (caloric testing): Caloric testing is a validated clinical tool to assess the vestibular system in each inner ear by stimulating the vestibulo-ocular reflex. A physician performs the test bedside using a caloric irrigation system. Warm water is introduced first, inducing reflexive nystagmus. Physicians should wait five minutes before performing the test on the other ear. The stimulus should be alternated to cold water to produce nystagmus again if indicated. Patient’s eye movements are measured after the caloric test in both ears and subsequently compared to normal values and each other [54]. This test can be used to differentiate between central and peripheral presentations of dizziness. Caloric testing was found to be 74.65% sensitive and 83.54% specific [53].

Vestibular function tests (vestibular evoked myogenic potential (VEMP)/rotational chair): There are two distinct types of VEMP testing: cervical VEMP and ocular VEMP. Cervical VEMP delivers vibrations to a patient wearing headphones to stimulate hair cells on the otoliths. The test measures the vestibulo-collic reflex by recording the inhibition of the sternocleidomastoid muscle after activation of the otolith hair cells. Ocular VEMP is measured through electrodes placed under the eyes. The stimulus can vary between bone-conducted and air-conducted sound to stimulate the vestibulo-ocular reflex. If there are diminished or absent reflexes in the VEMP test, there may be pathology present [52].

The stimulus in these tests can vary, which may make standardization of abnormalities more difficult when performing these tests. Rotational chair testing is a calibrated chair where a patient’s horizontal vestibulo-ocular reflex is tested using either sinusoidal harmonic acceleration or step testing. The head velocity is compared to the eye velocity in gain, time, phase, and symmetry. Rotational testing had a specificity of 78.48% and a sensitivity of 76.06% [53]. It is noted that rotational chair testing is best for bilateral vestibular dysfunction rather than unilateral deficits [55].

Audiometry: Pure-tone audiometry is a standardized test frequently used in clinics. Patients are exposed to low, medium, and high frequency tones. Every time the patient hears a sound, they will push a button. This measures if there is unilateral or bilateral hearing loss and at which frequencies. One study found that the reliability of the test is increased when insert earphones are used [56]. In those with migraine-associated dizziness, this test manifests as low-frequency mild hearing loss [57].

Imaging: Neuroimaging can be indicated for acute vertigo and dizziness. Cranial CT is most often the imaging of choice in the emergency department. However, CT may not detect smaller lesions or detect posterior cerebral artery strokes (sensitivity of 16% for the latter) [58]. MRI can be indicated to aid in the diagnosis of acute vestibular dysfunction [59]. One study found that MRI has lower sensitivity when compared to an oculomotor exam in acute settings [58]. Neuroimaging should be reserved to rule out dangerous, emergency conditions rather than to diagnose a specific vestibular disorder.

Diagnostic Criteria Summary

POTS is a chronic autonomic disorder characterized by an increase in heart rate when standing, while blood pressure is maintained. A consensus has been reached outlining the defining features to receive a diagnosis of POTS in adults and adolescents. For adults, this is defined as a rise in heart rate of at least 30 beats per minute within 10 minutes of standing. For adolescents, the rise in heart rate must be at least 40 beats per minute within 10 minutes of standing for diagnosis. There is also a lack of orthostatic hypotension. Other common symptoms when standing include generalized weakness, palpitations, and lightheadedness, which are relieved when the patient is supine. Symptoms must be present for at least three months before diagnosis [10].

VM diagnostic criteria are combined from the International Classification of Headache Disorders (ICHD) and the Barany Society. There must be at least five episodes with recurrent vestibular symptoms and a history of migraines, according to the ICHD. The episodes can last up to 72 hours and are rated between moderate and severe. The Barany Society classifies vestibular symptoms as spontaneous vertigo, visually induced vertigo, head-movement induced vertigo with nausea, or positional vertigo [60]. Studies have found that spontaneous vertigo is the most common vestibular symptom [61]. These diagnostic criteria focus on episodic VM. Patients can experience chronic VM. However, no specific distinction in diagnosis criteria has been made yet.

PPPD is a chronic vestibular disorder where symptoms of non-spinning vertigo, unsteadiness, or dizziness are present for at least three months. The symptoms last for hours at a time and are present on most days. There may or may not be specific triggers, but symptoms are worsened by an upright position, any active or passive motion, and moving visual stimuli. PPPD leads to significant functional impairment or distress. Before a PPPD diagnosis, many patients have a precipitating condition. The most common being peripheral or central vestibular disorders (around 30% or cases) and VM (around 20% of cases) [3]. 

Differentiating algorithms

Symptom Pattern Logic

There is a strong need for a symptom classification system based on the characteristics of the dizziness episodes. To that end, a practical and logical differentiation system can be developed from the time course and provoking factors of dizziness, and not solely based on the descriptors provided by the patient. For example, orthostatic dizziness would be diagnosed if the symptoms consistently follow the patient's standing position and resolve upon reclining, while associated with other symptoms characteristic of orthostatic intolerance phenotypes, such as POTS [10]. Episodic syndromes are generally characterized by discrete episodes (lasting minutes to hours) followed by symptom-free periods; vertigo or motion intolerance with migrainous symptoms (photophobia, phonophobia, nausea, or headache) would support a diagnosis of VM [4].

Chronic syndromes would typically involve dizziness that is present for most days over a period of months; these symptoms are frequently worsened by standing, movement, and visually complicated environments; this type of symptom profile is typical of PPPD and represents a process of maladaptive sensory weighting rather than a chronic peripheral lesion [3]. Notably, it is quite common for there to be overlap among the different diagnoses listed above, so the clinician should concentrate on identifying the dominant precipitant and temporal structure of the dizziness rather than focusing on assigning a specific diagnosis too quickly. The use of an orthostatic, episodic, chronic framework for reviewing the differential will provide a methodologically valid and replicable starting point for the development of a mechanistically directed evaluation.

Exam and Test Flow

A systematic and step-wise approach for ENT and neurological clinicians. A step-wise approach to examining and testing dizziness should begin with a bedside triage to identify common vestibular disorders, followed by assessing the physiological basis for orthostasis when there are indicators that the dizziness is related to presyncope. In both ENT and neurology settings, the initial decision point is to establish whether the clinical presentation is acute and possibly life-threatening (e.g., new focal neurological deficits, severe gait ataxia, persistent vertical nystagmus, or significant vascular risk factor), causing a rapid neurological evaluation and relevant imaging studies rather than an outpatient evaluation for dizziness. Once the clinical presentation has been determined to be non-acute, the clinician may proceed with a systematic otolaryngology and vestibular evaluation (otoscopy, hearing screening/ audiometry when clinically indicated, ocular motor evaluation, positional testing-e.g., Dix-Hallpike for BPPV), and apply syndrome-specific criteria when applicable.

When the clinical presentation includes episodic symptoms with migrainous accompaniments, applying VM diagnostic criteria and collaborating with the patient's neurologist is recommended [4]. When the clinical presentation involves chronic symptoms that are visually/motion-provoked, but a neurologic and vestibular examination is normal, applying the PPPD diagnostic criteria and referring the patient to a specialist for vestibular rehabilitation and/or behavioral therapy is recommended [3]. When the clinical presentation indicates that symptoms are posture-dependent or recumbency-responsive, the evaluation process should include standardized orthostatic vital signs and, when necessary, confirmatory autonomic evaluations (e.g., tilt-table evaluation) [10,49]. This sequential approach embodies a "test what you suspect" strategy and assures that testing follows the primary symptom provoker.

Pitfalls and Red Flags

Common pitfalls and red flags (misclassification of PPPD as peripheral vestibulopathy and failure to identify autonomic symptoms). One of the most common pitfalls is misclassifying PPPD as a chronic labyrinthitis or some other peripheral vestibulopathy because patients report experiencing "imbalance" each day; however, the neurologic and vestibular exams are usually normal, and the symptoms appear to be largely provoked by visual complexity and motion [3]. Another common error is to overlook autonomic symptoms-e.g., palpitations, heat intolerance, worsening symptoms with posture, improvement with recumbence-and to proceed directly to repeat vestibular testing or increasing vestibular testing without first obtaining standardized orthostatic vital signs, thereby delaying recognition of orthostatic intolerance and prolonging the patient's functional impairment [10].

Clinicians must be cautious about "ear symptom anchoring," where nonspecific aural fullness or subjective ear pressure prompts a premature diagnosis of Ménière disease when the overall clinical picture is more suggestive of VM or some other non-vestibular cause. Therefore, all red flags should be carefully queried since posterior circulation strokes may present primarily with vertigo and may be missed by early imaging studies, including false-negative MRIs of small strokes [58]. Additionally, transient vestibular syndromes can still be representative of cerebrovascular events, and clinicians' bedside assessments are necessary to prevent inappropriate validation or delayed escalation [59]. An effective algorithm, therefore, uses both trigger-based logic and careful red flag screening to minimize both benign mislabeling and dangerous under-triage.

Management strategies

Postural Orthostatic Tachycardia Syndrome (POTS)-Focused Interventions

Management of POTS differs for each individual, and treatment is multimodal with non-pharmacologic strategies first-line and pharmacologic therapy for severe and refractory cases. Fluid intake of 2-3 L/day and sodium intake of up to 10-12 g/day when appropriate should be considered to improve orthostatic symptoms. Short-term clinical decompensations can be treated with an acute intravenous (IV) infusion of up to two L of saline [62]. Compression garments such as waist-high compression stockings can reduce venous pooling [62]. Isometric physical counterpressure movements of large muscles (e.g., handgrip, squatting, leg crossing) can increase afterload and blood pressure. One study observed that graded exercise therapy in a semi-recumbent position to avoid upright posture, such as recumbent bike, swimming, and rowing, improved symptoms by increasing the aldosterone-to-renin ratio and decreasing heart rate [61]. These combined interventions offer a more effective therapy than simply pharmacologic interventions to lower heart rate alone [61]. Pharmacologic therapy, when appropriate consist of beta blockers, volume expanders, and midodrine. Ivabradine can lower sinus rate without affecting blood pressure. Pyridostigmine, a peripheral acetylcholinesterase inhibitor, can blunt orthostatic tachycardia. Fludrocortisone may be useful to increase sodium retention and expand plasma volume [62]. Beta-blockers may be used to reduce sinus tachycardia and palpitations in POTS. One study observed that low-dose oral propranolol attenuated tachycardia in POTS and elicited a better response than high-dose oral propranolol, which did not improve or may have worsened symptoms [5]. Midodrine is a drug metabolized to a peripheral alpha-1 agonist that can increase venous return and reduce orthostatic tachycardia. Both oral and intravenous saline can be used as volume expanders to improve symptoms for some patients in the short term [62].

Vestibular Migraine (VM) Treatments

Management of VM follows established migraine prevention protocols, as they have favorable responses to anti-migraine drugs [4]. This includes preventative treatment with divalproex sodium, sodium valproate, topiramate, metoprolol, propranolol, and timolol [63]. Lifestyle modification is recommended to avoid potential triggers when possible. These triggers may include menstruation, stress, lack of sleep, dehydration, and certain foods [4].

Persistent Postural-Perceptual Dizziness (PPPD) Treatment

When dealing with PPPD and chronic vestibular disorders, vestibular rehabilitation therapy can help patients feel balanced. Cognitive behavioral therapy (CBT) might help patients who have fears associated with falling or dizziness since it can reduce similar behavior in patients with anxiety disorders. Selective serotonin reuptake inhibitors (SSRI) and serotonin norepinephrine reuptake inhibitors (SNRI) may be treatment options for chronic functional dizziness and PPPD as they can reduce dizziness and unsteadiness [32].

Coordinated Care Model

Referrals to cardiology are recommended when diagnostic criteria for POTs are met, as outlined in guidelines, especially when symptoms interfere with daily living and further testing or medication is needed [62]. Referral to neurology is suggested when vestibular migraine is suspected based on symptoms of vertigo and migraine in accordance with diagnostic criteria [4]. Vestibular physical therapy is indicated when there is suspected PPPD due to persistent imbalance and heightened motion sensitivity [3].

Case vignettes

Case 1: Postural Orthostatic Tachycardia Syndrome (POTS)​​​​​​​ Misdiagnosed as Benign Paroxysmal Positional Vertigo (BPPV)

A 23-year-old female presents to her new PCP with recurrent episodes of dizziness for six months. She experiences dizziness mostly when transitioning from sitting to standing or after standing for prolonged periods of time. She also relates palpitations, difficulty sustaining focus, and fatigue. She reports that her dizziness gets better after lying down. Her previous PCP had diagnosed her with BPPV and had been treated with canalith repositioning maneuvers with little relief. On physical examination, her orthostatic vitals show an increase of 30 in heart rate from lying down to standing, and blood pressure remained similar. Testing with the Dix-Hallpike maneuver is negative. Head movement does not exacerbate symptoms. She is referred to an electrophysiology lab, where her tilt table testing is positive. Based on history and physical examination findings, the patient is diagnosed with POTS. She is recommended to increase fluid and salt intake, use compression garments, and start a structured recumbent exercise program. She is prescribed low-dose oral propranolol to manage palpitations. Over the next few months, she notices a significant improvement in dizziness and quality of life.

*Case 2: Vestibular Migraine (VM)​​​​​​​* *Mistaken for Meniere’s*

A 40-year-old female presents with episodes of vertigo that last four to five hours. She reports feeling nauseous during these episodes, with pulsatile headaches on the right side of her forehead, and discomfort in bright lights. She also experiences right ear fullness but denies any hearing loss in either ear. Her past medical history is positive for Meniere’s disease. Although she was put on dietary sodium restriction and prescribed a diuretic, her symptoms persist. On physical examination, Weber and Rinne’s tests are negative. Additionally, testing with audiometry is normal. During oculomotor evaluation, there is sustained vertical nystagmus. The patient is diagnosed with VM, and her treatment plan is adjusted according to the diagnosis. She is started on migraine prophylaxis with topiramate and counseled on migraine trigger modification strategies. The frequency and severity of the vertigo and headaches improve over the next several months.

Case 3: Persistent Postural-Perceptual Dizziness​​​​​​​ (PPPD) Mislabeled as Chronic Labyrinthitis

A 45-year-old female presents with a chief complaint of persistent non-vertiginous dizziness and imbalance for over a year after an illness. She relates dizziness lasting for hours on most days, with worsening symptoms in busy environments like the mall or when looking down at her phone at work. She was previously told by her clinician that her symptoms were related to chronic labyrinthitis. Her past medical history is positive for vestibular migraine, which is well controlled. On physical examination, orthostatic vital signs show no changes. Neurological and vestibular examinations are normal. After a diagnosis of PPPD is made, referrals to vestibular therapy and cognitive behavioral therapy are given, and an SSRI is initiated. Gradually, there is reduced motion sensitivity and improved functioning.

Discussion

The authors emphasize that chronic dizziness in otolaryngology (ENT) practice cannot be fully understood by simply fitting it into standard diagnostic categories (silos) based on the physical site where dizziness occurs. It needs to be seen as a symptom that has many overlapping characteristics. For example, POTS, VM, and PPPD all describe individuals who experience dizziness (or what they call "imbalance") or brain fogginess ("brain fog"), both of which can be caused by upright posture, movement, or visual complexity. However, each of these conditions has different primary mechanisms. For example, POTS is due to an abnormality of the autonomic nervous system (the system that governs involuntary functions of the body such as heart rate, digestion, etc.), Vestibular Migraine is due to a dysfunction of the parts of the brain involved in processing pain and nausea, and PPPD is thought to be due to a maladaptive process of how the body registers and interprets sensory information.

Mechanistically, there are two reasons that a patient might experience worsening of symptoms in both head motion and standing. First, vestibulo-autonomic integration describes the physiological reason why a patient's symptoms can worsen at both the time of head motion and at the time of standing. Second, central sensory amplification and maladaptive sensory reweighting explain why triggers for symptoms can be broader and disabilities sustained over a longer period of time than expected for disorders originating from migraine circuitry, autonomic dysregulation, or functional neuro-otologic adaptation [14,20,32].

Clinically, this supports a trigger-anchored approach to diagnosis. Symptom timing and context will guide the clinician in determining if the patient meets criteria for a particular condition. Therefore, posture-dependent symptoms that improve with recumbency suggest orthostatic intolerance, whereas discrete episodic vertigo with migrainous features and chronic visually-induced unsteadiness are suggestive of Vestibular Migraine and PPPD, respectively [3-4]. Recognizing this spectrum is necessary to avoid attributing all dizziness to peripheral vestibular pathology and to provide a closer match between diagnostic reasoning and underlying physiology.

In terms of the impact of dysautonomia on ENT clinicians, ENT dizziness clinics routinely encounter patients with non-specific symptom reports and those with unrevealing vestibular testing. Furthermore, prior treatments usually target presumed vestibular causes without considering the effects of orthostatic physiology. As a result, patients remain disabled and continue to utilize health services [2,10]. Thus, the manuscript's focus on identifying patients' orthostatic triggers and using bedside orthostatic vital signs is particularly relevant. A simple, standardized active-stand protocol (supine rest followed by repeated standing heart rate and blood pressure measurements) can help identify patients with orthostatic hypotension or orthostatic tachycardia phenotypes that could otherwise be mislabeled as vestibular migraine or "functional dizziness," especially if the primary complaints are non-spinning dizziness, fatigue, or cognitive symptoms [10,43-44].

In addition, this approach may result in a reappraisal of "visually-induced dizziness" and cognitive exhaustion in the context of possible cerebrovascular dysregulation and neurovascular coupling abnormalities in POTS, rather than as evidence of primary labyrinthine disease [11]. Therefore, incorporating autonomic thinking into the ENT dizziness evaluation will increase diagnostic validity and strengthen interdisciplinary referrals without devaluing the importance of vestibular assessment.

Diagnostic tools currently available in the field play a role in misclassification and delayed diagnosis. Vestibular testing can be useful, but it is imperfect. Vestibular migraine may exhibit abnormalities across all modalities of testing; however, the sensitivity of testing varies, and normal results do not rule out centrally mediated or autonomic contributors to dizziness. Imaging is best used to rule out dangerous etiologies and not to define a chronic dizziness syndrome [53,58]. From an autonomic perspective, tilt-table testing can provide physiologic proof of the presence of orthostatic intolerance and reproduce symptoms, but may be limited by lack of availability and/or eligibility requirements, thus preventing some patients from having a definitive pathway to diagnosis despite experiencing clinically significant orthostatic intolerance [49-50]. These limitations emphasize the clinical value of (1) structured, trigger-based history-taking (orthostatic vs. positional vs. sensory-environmental), (2) standardized, correct performance of orthostatic vitals in the clinic setting, and (3) coordinated care pathways that refer patients to appropriate treatment and therapy based on the suspected mechanism(s) rather than waiting for a single "rule-out" test [42-43].

Therefore, there is clearly a need for prospective studies of integrated diagnostic strategies in ENT populations. Future studies should determine whether the combination of standardized orthostatic vitals with vestibular examination/testing and symptom-trigger phenotyping leads to augmented diagnostic validity, shorter time-to-treatment, and greater predictability of response to intervention based on mechanism [3,10]. The manuscript's future directions furnish actionable research targets: autonomic biomarkers (e.g., heart rate variability, plasma norepinephrine), integrated vestibulo-autonomic testing paradigms, and home/telemedicine orthostatic monitoring to measure the physiologic responses of patients in their environment [64-66]. Prospectively verifying these approaches could represent a shift from symptom-based classification to physiology-based diagnostic pathways that more accurately reflect the overlap of chronic dizziness syndromes and may ultimately result in better outcomes for patients through enabling earlier, targeted treatment.

Future directions

Biomarkers of Autonomic Dysfunction

Advances in evaluating chronic dizziness have increased emphasis on biomarkers of autonomic impairment, reflecting a shift towards objective physiological measurement rather than symptom reliance alone. These markers include heart rate variability (HRV), blood pressure monitoring, plasma norepinephrine levels, sudomotor testing, and small fiber neuropathy assessment [66]. Ziemssen and Siepmann’s dysautonomia perspective views it as a spectrum of measurable neuro-cardiovascular abnormalities, challenging the notion that orthostatic syndromes are diagnoses of exclusion. Reduced HRV reflects impaired parasympathetic modulation and has been associated with dysautonomia symptoms [67]. Wang’s study suggests that diminished vagal tone can predispose patients to exaggerated sympathetic responses during postural stress, causing disproportionate dizziness with minimal triggers.

Focusing on symptoms alone could’ve led to misdiagnosis. Elevated standing plasma norepinephrine (>600 pg/mL) supports a hyperadrenergic POTS phenotype [68]. By distinguishing a hyperandrogenic subtype, Lei’s study further emphasizes the importance of biomarkers in conjunction with identifying symptoms to accurately diagnose patients. Additionally, small fiber neuropathy has been linked to POTS, causing symptoms such as allodynia, hyperesthesia, restless leg syndrome, as well as cardiac manifestations that include syncope, palpitations, and orthostatic hypotension [69]. Levine implies that diagnosing POTS beyond cardiovascular symptom criteria can include testing for peripheral neuropathic processes that may underlie the syndrome’s sensory and autonomic symptoms. Although no single biomarker is diagnostic, autonomic profiling can improve diagnosis and guide targeted therapy.

Integrated Vestibulo-Autonomic Testing

Integrated vestibulo-autonomic testing is a diagnostic modality that recognizes vestibular and autonomic systems as interconnected through the vestibulo-sympathetic reflex [12]. Yates’s work provides a basis for the frequent coexistence of dizziness and orthostatic symptoms, indicating that the overlap is not a coincidence. Simultaneous assessment of vestibular function (vHIT, VEMP, caloric testing) and autonomic responses (heart rate and blood pressure during tilt or postural challenge) can help distinguish primary vestibular disorders from orthostatic intolerance presenting with secondary dizziness [64]. Tarnutzer demonstrates that the correlation between symptom onset and physiologic change is more important for diagnosis than symptoms alone. Abnormal autonomic responses to vestibular stimulation may contribute to symptom overlap between vestibular migraine, PPPD, and dysautonomia [20]. This perspective implies that these conditions share pathophysiologic origin, challenging diagnosis using individual criteria and supporting a spectrum of chronic dizziness. An integrated physical examination can be implemented by incorporating orthostatic vital signs and tilt-table testing into the evaluation of dizziness in ENT clinics.

Telemedicine for Orthostatic Evaluation

Telemedicine-based orthostatic assessment has become increasingly popular, following the expansion of remote healthcare during COVID-19. Home-based active stand tests, wearable heart rate monitors, and remote blood pressure devices allow clinicians to evaluate orthostatic tachycardia and related symptoms in real time [65]. Gabriel’s study highlights that real-time data collection may enhance diagnostic validity, as vital signs are more accurate when they’re obtained in an everyday environment rather than a medical office. POTS guidance now supports remote orthostatic vitals when tilt-table testing is unavailable, given standardized protocols are followed [70]. Such updated diagnostic standards provide patients with more medical accessibility. Patients with POTS usually require chronic management from multiple specialists, so telehealth follow-up is valuable for these populations. Not only does continuous monitoring improve the continuity of care, but it can also modify therapy based on symptom updates. As digital health programs advance, autonomic monitoring is improving the diagnosis and treatment of POTS.

## Conclusions

This review outlines an evidence-based approach to distinguishing the symptoms of orthostatic dizziness due to dysautonomia from those of VM and PPPD. The approach is based on three elements: timing, triggers, and objective bedside physiologic measures, rather than relying on vague descriptions of “dizziness.” When patients experience postural-dependent dizziness that improves upon recumbency, ENT physicians should specifically consider presyncope and orthostatic intolerance and obtain standardized orthostatic vital signs to determine if POTS or orthostatic hypotension phenotypes are present. While episodic vertigo with migraine features and persistent visually-induced unsteadiness with motion sensitivity are the primary clinical indicators of VM and PPPD, respectively, identifying these patterns facilitates targeted vestibular, neurologic, and rehabilitative treatment strategies.
